# Drug-Coated Balloon PCI in Different Plaque Morphologies: A Narrative Review

**DOI:** 10.3390/biomedicines13061472

**Published:** 2025-06-14

**Authors:** Flavius-Alexandru Gherasie, Raluca Ciomag (Ianula), Luana-Maria Gherasie

**Affiliations:** 1 Department of Cardiology, University of Medicine and Pharmacy “Carol Davila”, 050474 Bucharest, Romania; 2 Emergency Clinical Hospital Dr. Bagdasar-Arseni, 050474 Bucharest, Romania; 3 “Prof. Dr. D. Hociota” Institute of Phonoaudiology and Functional ENT Surgery, 061344 Bucharest, Romania; luana-maria.bujor@drd.umfcd.ro

**Keywords:** drug-coated balloon, paclitaxel-coated balloon, sirolimus-coated balloon, in-stent restenosis, de novo lesions, coronary artery disease, percutaneous coronary intervention, vascular remodeling, plaque morphology

## Abstract

The evolution of percutaneous coronary intervention (PCI) has led to significant advances in drug-coated balloon (DCB) technology, offering a stent-free alternative for treating coronary artery disease. While paclitaxel-coated balloons (PCBs) have been the standard, sirolimus-coated balloons (SCBs) are emerging as a viable alternative with distinct pharmacokinetic and clinical benefits. This review explores the mechanisms of action of paclitaxel and sirolimus, their impact on different plaque morphologies, and the clinical implications of DCB selection. Paclitaxel facilitates positive vascular remodeling and is particularly effective in fibrotic and lipid-rich plaques, but its poor penetration in calcified lesions remains a limitation. Sirolimus, with its homogeneous tissue distribution and anti-inflammatory properties, is better suited for unstable, lipid-rich, and inflammatory plaques, where it promotes plaque stabilization. Recent randomized trials and meta-analyses have compared SCBs vs. PCBs in both de novo lesions and in-stent restenosis, showing non-inferior outcomes. Additionally, DCBs demonstrate comparable efficacy to DES in small vessel disease, reducing the need for permanent metallic scaffolds. This review summarizes the current evidence on DCB selection based on plaque characteristics and highlights areas for further investigation in personalized PCI strategies. Given the narrative review design, the authors conducted a comprehensive literature search using databases such as PubMed and MEDLINE. Keywords included “drug-coated balloon”, “paclitaxel-coated balloon”, “sirolimus-coated balloon”, “in-stent restenosis”, and “plaque morphology”. Studies were selected based on relevance, including randomized controlled trials, registries, and meta-analyses. No formal inclusion/exclusion criteria or systematic screening were applied due to the nature of narrative synthesis.

## 1. Introduction

Percutaneous coronary intervention (PCI) has undergone significant technological evolution over the past three decades, transitioning from balloon angioplasty to bare-metal stents (BMSs), drug-eluting stents (DESs), and most recently, drug-coated balloons (DCBs). While DES remains the cornerstone of revascularization for most coronary lesions, emerging data have highlighted specific clinical scenarios where DCBs offer distinct advantages, particularly in small vessels, in-stent restenosis (ISR), and patients at high bleeding risk.

DCBs deliver antiproliferative agents without leaving behind a permanent implant, allowing for vessel healing without the long-term complications associated with stent platforms. Recent advances in coating technologies have introduced two dominant types of DCBs—paclitaxel- and sirolimus-coated balloons—each with unique drug properties, elution kinetics, and tissue interactions.

This review focuses on the performance of these two types of DCBs in relation to plaque morphology, namely lipid-rich, fibrotic, and calcified lesions. We aim to provide a narrative synthesis of the available clinical and mechanistic evidence, exploring how plaque composition may influence the choice of DCB platform and procedural strategy. In addition to summarizing clinical evidence, this review aims to offer a practical, morphology-based framework to guide drug-coated balloon selection. By linking pharmacological mechanisms with specific plaque characteristics, the review provides a novel interpretive lens to aid clinical decision making and highlight gaps for future investigation.

**Figure 1:**
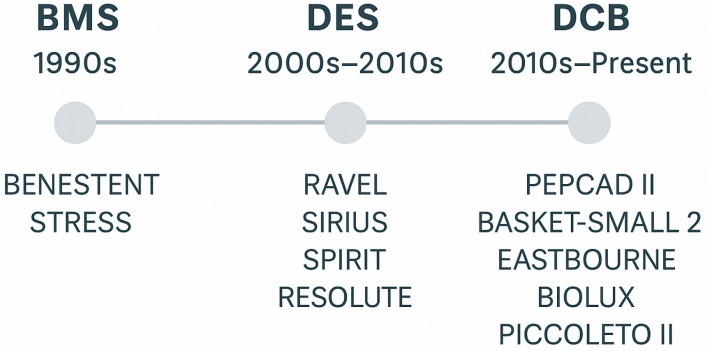
Historical roadmap of PCI development, illustrating the transition from bare-metal stents (BMSs) to drug-eluting stents (DESs), and more recently to drug-coated balloons (DCBs), along with representative landmark trials at each stage.

The limitations of BMS led to the development of drug-eluting stents (Figure 1). These stents are coated with antiproliferative drugs, such as sirolimus or paclitaxel, which are gradually released to inhibit neointimal hyperplasia [1,2,3,4,5]. The introduction of drug eluting stent (DES) significantly reduced the rates of in-stent restenosis compared to BMS. For instance, studies demonstrated that first-generation DES had restenosis rates as low as 3.2%, a substantial improvement of up to over 30% observed with BMS [6]. Advancements led to second-generation DES like the everolimus-eluting Xience [7] and zotarolimus-eluting Endeavor [8]. These stents featured improved biocompatible polymers and thinner struts, further decreasing restenosis rates. Clinical trials demonstrated target lesion revascularization rates of 5.3% for DES compared to 10.3% for BMS [9]. Recent innovations focus on third-generation DES and ultrathin strut designs and biodegradable polymers to enhance vascular healing and reduce restenosis. These stents aim to minimize arterial injury and promote rapid endothelialization, leading to even lower restenosis rates [10,11].

## 2. Evolution of PCI and the Rise of Drug-Coated Balloons

**Figure 2:**
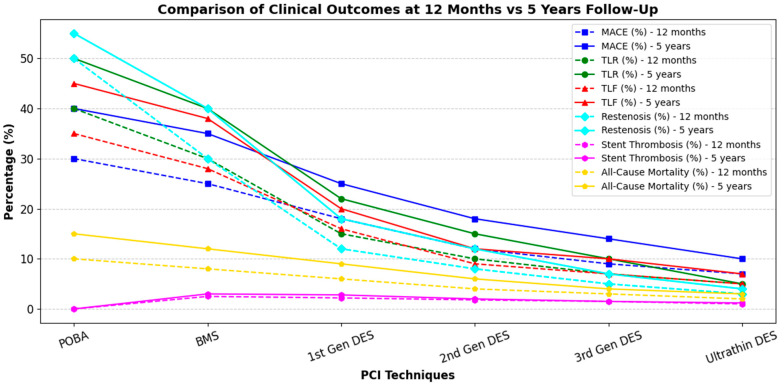
Graphical comparison of clinical outcomes at 12 months’ vs. 5 years’ follow-up (MACE, TLR, TLF, restenosis, stent thrombosis, and death) across different PCI techniques, from POBA to ultrathin DES. MACE: major adverse cardiac event; TLR: target lesion revascularization; TLF: target lesion failure.

The evolution of percutaneous coronary intervention techniques has led to significant improvements in clinical outcomes over time. A comparative analysis of 12-month and 5-year follow-up data across different PCI strategies—POBA, BMS, First-, Second-, and Third-DES, and Ultrathin DES—reveals key trends in MACE, target lesion revascularization (TLR), target lesion failure (TLF), restenosis, stent thrombosis, and mortality (Figure 2).

The limitations of BMS, particularly high rates of restenosis, led to the introduction of first- and second-generation DES, which reduced neointimal hyperplasia through local drug delivery [12,13,14,15]. However, DES implantation can still result in complications such as delayed endothelial healing, late stent thrombosis, and the need for prolonged dual antiplatelet therapy (DAPT).

DCBs emerged as a stent-free alternative, combining the benefits of balloon angioplasty with localized drug delivery. Unlike DES, DCBs do not leave a metallic scaffold, which may allow for more natural vascular healing and less need for extended DAPT. These properties make DCBs particularly appealing in complex clinical contexts, such as ISR, small vessels, bifurcations, and high-bleeding-risk patients [16,17].

Given the growing clinical use of DCBs, understanding how plaque morphology influences their pharmacological effectiveness and clinical performance is increasingly important. The remainder of this review will explore the differential effects of paclitaxel and sirolimus DCBs in the context of specific plaque types and lesion characteristics.

## 3. The Advantages of Drug-Coated Balloons over Stents

One of the key benefits of drug-coated balloons is their ability to deliver antiproliferative drugs without leaving behind a permanent implant, offering several advantages over drug-eluting stents.

DCBs preserve vascular physiology by eliminating the need for a metallic scaffold. Unlike DES, which leave a permanent stent in the artery, DCBs deliver the drug directly to the vessel wall and allow the artery to heal naturally, maintaining its elasticity and function.

Another significant advantage is the reduced risk of late stent thrombosis. First-generation DESs were associated with an increased incidence of late and very late thrombosis due to delayed endothelialization and chronic inflammation caused by the stent struts. By avoiding a permanent implant, DCBs minimize this risk, making them a safer long-term option in select patients. DCBs also offer the benefit of shorter dual antiplatelet therapy (DAPT) duration, which is particularly important for high-bleeding-risk patients. Since no foreign material remains in the vessel, the requirement for prolonged DAPT is reduced, lowering the risk of bleeding complications while still maintaining effective restenosis prevention.

Additionally, DCBs are highly effective in small vessels and bifurcations, where stenting poses significant challenges. Small coronary arteries have higher restenosis rates with stents due to their limited lumen size, and bifurcation lesions often require complex techniques to prevent side-branch occlusion. DCBs provide homogeneous drug delivery without the mechanical constraints of stents, making them an attractive option in these anatomically challenging scenarios [18].

By offering a stent-free approach with comparable efficacy, DCBs represent an important advancement in PCI, particularly for patients where avoiding a permanent implant is beneficial.

With growing evidence supporting their efficacy, DCBs are now being investigated for de novo lesions. Trials like BASKET-SMALL 2 and PICCOLETO II have shown that DCBs can be a viable alternative to DES in small vessel disease, with comparable long-term outcomes and lower revascularization rates [19,20].

## 4. Drug Carriers for Paclitaxel and Sirolimus DCBs

The balloon used in DCB therapy is typically compliant or semi-compliant, designed to expand the vessel during inflation while ensuring optimal drug delivery to the arterial wall. As the balloon inflates, it temporarily widens the vessel, allowing the drug-coated surface to come into direct contact with the endothelium.

The active drug in most DCBs is paclitaxel, a potent antiproliferative agent that inhibits smooth muscle cell proliferation, the primary cause of restenosis. Paclitaxel is highly lipophilic, enabling efficient penetration into the vessel wall, where it remains active for an extended period to prevent neointimal hyperplasia. The standard paclitaxel dose used in DCB therapy typically ranges from 2 to 3.5 μg/mm^2^ [21].

To facilitate drug transfer, paclitaxel is combined with a carrier substance, which plays a critical role in ensuring efficient absorption and retention of the drug within the vessel wall. Since paclitaxel is inherently hydrophobic, the carrier helps improve its solubility and adhesion to the endothelial surface, ensuring maximum therapeutic efficacy during balloon inflation. This precise drug delivery mechanism allows for effective restenosis prevention while eliminating the need for a permanent stent. These carriers influence drug solubility, tissue penetration, and clinical efficacy (Table 1). Paclitaxel-coated balloons (PCBs) use carriers such as Iopromide, Butyryl-trihexyl-citrate (BTHC), and Urea to enhance drug adhesion and transfer. Iopromide, a hydrophilic contrast agent, is used in the SeQuent Please DCB and provides rapid paclitaxel transfer within 30–60 s, though it carries a risk of distal embolization. BTHC, a lipophilic excipient used in the Pantera Lux DCB, allows for better retention in tissues, requiring 45–60 s for optimal drug transfer [22,23]. Urea, used in the Prevail DCB, enhances drug diffusion due to its hydrophilic nature, allowing rapid transfer within 30 s but leading to faster drug washout compared to lipophilic carriers [24].

Sirolimus-coated balloons (SCBs) require excipients to improve drug penetration and sustain therapeutic action due to sirolimus’ lower tissue permeability. Shellac, a hydrophobic carrier used in the Magic Touch DCB, ensures controlled release and prolonged drug retention, requiring at least 60 s for effective transfer [25]. Urea, used in the Selution SLR DCB, facilitates rapid sirolimus diffusion but may result in shorter tissue retention. Butyryl-trihexyl-citrate, also adapted for sirolimus in the Virtue DCB, enhances adhesion and prolongs retention, ensuring extended therapeutic action. Emerging phospholipid-based carriers, seen in experimental sirolimus DCBs, mimic natural cell membranes to improve absorption and sustain sirolimus release [26,27].

Clinically, paclitaxel DCBs have been widely studied and are effective in reducing restenosis but are associated with potential embolization and delayed healing. Sirolimus DCBs, with more controlled release profiles, may offer advantages in vessel healing, reducing inflammation, and lowering long-term adverse events. While studies indicate comparable outcomes in terms of late lumen loss, sirolimus DCBs might be preferable for high-risk lesions due to their better endothelial recovery profiles.

The review includes data from multiple DCB platforms (e.g., SeQuent Please, Magic Touch, Pantera Lux), which differ in carrier type, drug kinetics, and inflation duration. These variations can influence clinical outcomes, yet head-to-head data remain limited. The authors acknowledge this device heterogeneity and recommend that future trials compare outcomes across platforms to optimize DCB selection based on lesion characteristics.

## 5. DCB PCI for De Novo Lesions

Drug-coated balloons have emerged as a promising alternative to drug-eluting stents for the treatment of de novo coronary lesions, particularly in small vessel disease. Several studies have evaluated the efficacy and safety of DCBs in this context, including the BASKET-SMALL 2 and PICCOLETO II trials.

The BASKET-SMALL 2 trial was a randomized, noninferiority study that included 758 patients with de novo lesions in small coronary arteries (diameter < 3 mm). Participants were assigned to receive either DCB angioplasty or implantation of a second-generation DES. The primary endpoint was a composite of major adverse cardiac events, including cardiac death, nonfatal myocardial infarction, and target vessel revascularization, assessed at 12 months. The results demonstrated that DCBs were noninferior to DESs, with both groups experiencing a 15% rate of MACE at 12 months (hazard ratio [HR]: 0.99; 95% confidence interval [CI]: 0.68–1.45; *p* = 0.5). These findings persisted at the 3-year follow-up, supporting the long-term safety and efficacy of DCBs in small vessel de novo lesions [19].

The PICCOLETO II trial was a randomized study comparing a novel paclitaxel-coated DCB to an everolimus-eluting stent (EES) in patients with de novo lesions in small coronary vessels. The primary endpoint was late lumen loss (LLL) at 6 months, with secondary endpoints including MACE. The study found that the DCB group had significantly lower LLL compared to the EES group, indicating superior angiographic outcomes. Additionally, the DCB group demonstrated noninferiority in terms of MACE at 12 months, suggesting that DCBs may offer comparable clinical outcomes to DESs in this patient population.

Several meta-analyses and studies have evaluated the efficacy and safety of drug-coated balloons in treating de novo coronary lesions, offering insights into their clinical outcomes compared to other interventions.

**Figure 3:**
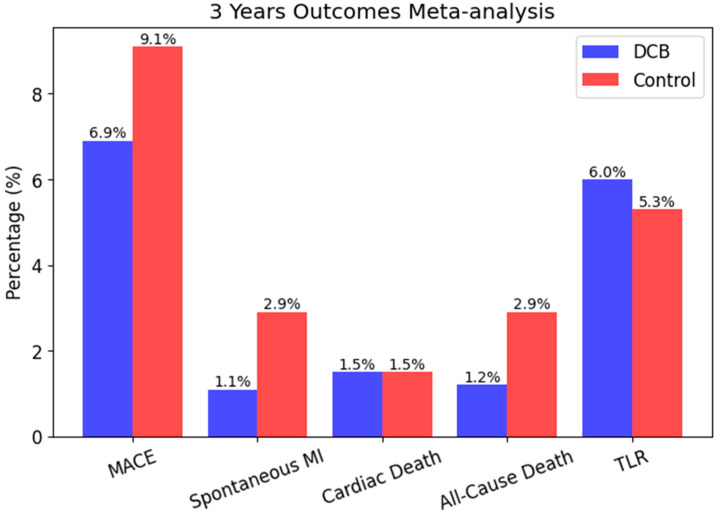
Graphical representation of 3-year outcomes meta-analysis (MACE, Spontaneous MI, Cardiac/All-cause Death, and TLR) across the studies BELLO, PEPCAD I, PEPCAD China SVD, PICCOLETO, RESTORE SVD China, BASKET-SMALL 2, DEBUT, IN.PACT Falcon, and Biolux P-I. Detailed breakdown of the control groups: control Group with BMS PCI: PEPCAD I, PEPCAD China SVD, DEBUT; control group with DES PCI: PICCOLETO, RESTORE SVD China, BASKET-SMALL 2, BELLO; control group with POBA: IN.PACT Falcon, Biolux P-I.

In a comprehensive meta-analysis which included fourteen randomized controlled trials with a total of 2483 patients, nine of them evaluated de novo lesions, BELLO (Balloon Elution and Late Loss Optimization), PEPCAD I (Paclitaxel-Eluting PTCA-Balloon Catheter in Coronary Artery Disease), PEPCAD China SVD, PICCOLETO (Paclitaxel-Coated Balloon Versus Drug-Eluting Stent During PCI of Small Coronary Vessels), RESTORE SVD China, BASKET-SMALL 2, DEBUT (Drug-Coated Balloon in Patients With High Bleeding Risk), IN.PACT Falcon, and Biolux P-I. The findings indicated that DCBs were associated with similar minimal lumen diameter (MLD), diameter stenosis, and binary restenosis rates compared to control treatments, with a lower late lumen loss. Importantly, there was no significant difference in target lesion revascularization (TLR) rates between DCBs and control strategies, suggesting that DCBs are a viable alternative for de novo coronary lesions [28,29,30] (Figure 3).

In 19 studies including 3643 patients with de novo coronary artery disease, outcomes between DCB and DES were compared. The analysis revealed that DCBs were associated with a significantly lower incidence of myocardial infarction (MI) and all-cause mortality compared to DES, highlighting the potential benefits of DCBs in specific patient populations [30].

These studies collectively support the use of DCBs as a safe and effective alternative to DES, particularly in specific clinical scenarios such as small vessel disease and patients at high bleeding risk. However, patient selection and lesion characteristics remain crucial factors in determining the optimal revascularization strategy (Table 2).

## 6. Paclitaxel vs. Sirolimus: Differences and Clinical Applications

When choosing between paclitaxel and sirolimus for drug-coated balloon therapy or stent-based interventions, it is essential to understand their distinct mechanisms of action and vascular effects. Both drugs are effective in preventing restenosis, but their impact on vascular remodeling and endothelial healing differs, influencing their optimal use in different clinical scenarios.

Understanding these differences helps guide the selection of the appropriate drug for specific lesions and patient conditions. Paclitaxel works by stabilizing microtubules, which inhibits smooth muscle cell proliferation and migration. This leads to a reduction in neointimal hyperplasia, the primary cause of restenosis. Additionally, paclitaxel has a unique advantage in that it promotes positive vascular remodeling, resulting in lumen enlargement and improved vessel patency. This effect is particularly beneficial in preventing late vessel narrowing after angioplasty or stent placement. In contrast, sirolimus inhibits the mechanistic target of rapamycin (mTOR) pathway, a key regulator of cell proliferation, growth, and survival. By blocking this pathway, sirolimus prevents smooth muscle cell overgrowth and reduces neointimal formation, effectively lowering restenosis rates. However, unlike paclitaxel, sirolimus does not promote positive remodeling. Instead, its primary effect lies in regulating matrix remodeling and cellular growth, which may lead to more controlled and stable vascular healing over time [31].

The difference in their effects on lesions further supports their distinct clinical applications. Paclitaxel is particularly effective at reducing neointimal hyperplasia while also promoting lumen enlargement, making it a strong choice in coronary interventions where maintaining long-term vessel patency is crucial. This remodeling effect is one of the reasons paclitaxel is frequently used in treating in-stent restenosis and small vessels, where late narrowing is a concern. Sirolimus, while equally effective in preventing smooth muscle cell proliferation, does not induce the same degree of vessel expansion. Instead, it is more focused on inhibiting excessive cellular growth and controlling matrix remodeling. Beyond their direct effects on the vessel wall, sirolimus has additional benefits in modulating immune responses and enhancing endothelial healing, contributing to better long-term vascular stability. This makes it a favorable choice for patients with complex lesions or a high risk of restenosis.

## 7. When to Choose Paclitaxel vs. Sirolimus Drug-Coated Balloon?

When choosing between paclitaxel and sirolimus, the clinical context is essential. Paclitaxel is preferred in cases where positive vascular remodeling is needed, such as in small vessel disease or in-stent restenosis, where preserving lumen size is critical. It is also beneficial in situations that require rapid drug transfer since paclitaxel is quickly absorbed into the vessel wall. On the other hand, sirolimus is more suitable for lesions requiring controlled vascular healing, particularly in complex cases where limiting inflammation and promoting endothelial recovery are priorities. It is also the preferred option for patients at high risk of restenosis, where a broader effect on neointimal suppression may be advantageous [32].

In conclusion, while both drugs effectively prevent restenosis, paclitaxel stands out for its positive remodeling effects, making it the drug of choice when lumen enlargement is crucial. Sirolimus, with its broader influence on vascular healing and matrix remodeling, is more suitable for complex lesions and cases where long-term vessel stability is the primary concern. The choice between paclitaxel and sirolimus should be guided by the specific characteristics of the lesion and the overall clinical needs of the patient.

## 8. Antithrombotic Therapy in DCB-Based PCI

A major clinical advantage of DCB-based PCI is the potential to reduce antithrombotic therapy burden, especially in high-bleeding-risk patients. Unlike DESs, which typically require prolonged dual antiplatelet therapy (DAPT), DCBs may permit shorter DAPT or P2Y12 inhibitor monotherapy protocols [33]. In patients with stable coronary artery disease undergoing DCB-only interventions, a shorter duration of dual antiplatelet therapy is generally recommended, typically around 1 month. For those presenting with acute coronary syndromes, current guidelines advise a longer DAPT duration, usually 12 months, regardless of whether the treatment involves a drug-coated balloon or a drug-eluting stent. However, in patients at high risk of bleeding, emerging evidence supports the consideration of shorter DAPT durations. This is particularly relevant in patients with atrial fibrillation, who require oral anticoagulation. In such cases, minimizing additional antiplatelet therapy is crucial. A recent review has outlined optimized strategies for PCI in AF patients, including DCB use to avoid triple therapy [34].

## 9. Paclitaxel and Sirolimus in Different Plaque Morphologies

Both paclitaxel and sirolimus exert potent antiproliferative effects, but their behavior varies depending on the composition of lipidic, fibrotic, and calcified plaques. Understanding these differences helps optimize treatment strategies for coronary artery disease (Table 3).

### 9.1. Lipid-Rich Plaques

Paclitaxel stabilizes lipid-rich plaques by reducing inflammation and thickening the fibrous cap, which lowers the risk of plaque rupture. Studies have shown that paclitaxel-coated balloons effectively reduce the lipid core burden index (LCBI), indicating plaque stabilization and improved vessel integrity. This makes paclitaxel particularly beneficial in treating lipid-rich plaques that are prone to rupture and subsequent cardiovascular events [35]. However, paclitaxel has limitations related to its lipophilicity and crystalline formulation, which may lead to non-uniform distribution in vessel walls and a risk of distal embolization. Consequently, in lipid-rich or unstable plaques, these limitations could theoretically pose concerns regarding inflammation, healing, and overall vessel wall stability.

In contrast, sirolimus, has a favorable pharmacokinetic profile, characterized by homogeneous vessel wall penetration and reduced inflammatory response, makes SEBs particularly appealing for more complex lesion morphologies, including lipid-rich plaques, thin-cap fibroatheromas, and inflammatory lesions. Such plaques are prone to rupture and associated with heightened inflammation, rendering sirolimus potentially superior in stabilizing these lesions and ensuring sustained therapeutic benefits. It lowers inflammatory markers such as IL-6 and TNF-α while increasing collagen content, which helps reinforce plaque stability. Additionally, sirolimus-coated balloons have shown efficacy in inhibiting macrophage proliferation and limiting lipid accumulation, which slows plaque progression and enhances vessel healing [36].

### 9.2. Fibrotic Plaques

Paclitaxel is highly effective in fibrotic plaques, where it inhibits smooth muscle cell proliferation, preventing excessive scar tissue formation and reducing neointimal hyperplasia. Studies have shown that paclitaxel significantly decreases restenosis rates in fibrotic plaques, limiting the excessive growth of smooth muscle cells that contribute to luminal narrowing [37]. Sirolimus also impacts fibrotic plaques, but it works primarily by modulating extracellular matrix remodeling rather than directly reducing smooth muscle proliferation. It has been shown to reduce fibrosis and enhance diastolic function, particularly in conditions characterized by excessive collagen deposition. In heart transplant recipients, everolimus was associated with a notable decrease in myocardial fibrosis, suggesting that sirolimus may help regulate fibrotic plaque progression as well [38].

### 9.3. Calcified Plaques

Calcified plaques present a challenge for drug penetration; generally plaque modification techniques prior to DCB angioplasty are needed. The dense, rigid nature of calcified plaques poses a significant barrier to the penetration of paclitaxel, leading to suboptimal drug distribution within the arterial wall. This limitation can result in reduced effectiveness of paclitaxel DCBs in heavily calcified lesions. Sirolimus’ effect on calcified plaques is less well-documented, but its ability to regulate inflammatory pathways and inhibit excessive vascular cell proliferation suggests that it may play a role in treating calcified lesions, particularly those with an active inflammatory component. However, its efficacy in fully calcified segments remains unclear.

**Figure 4:**
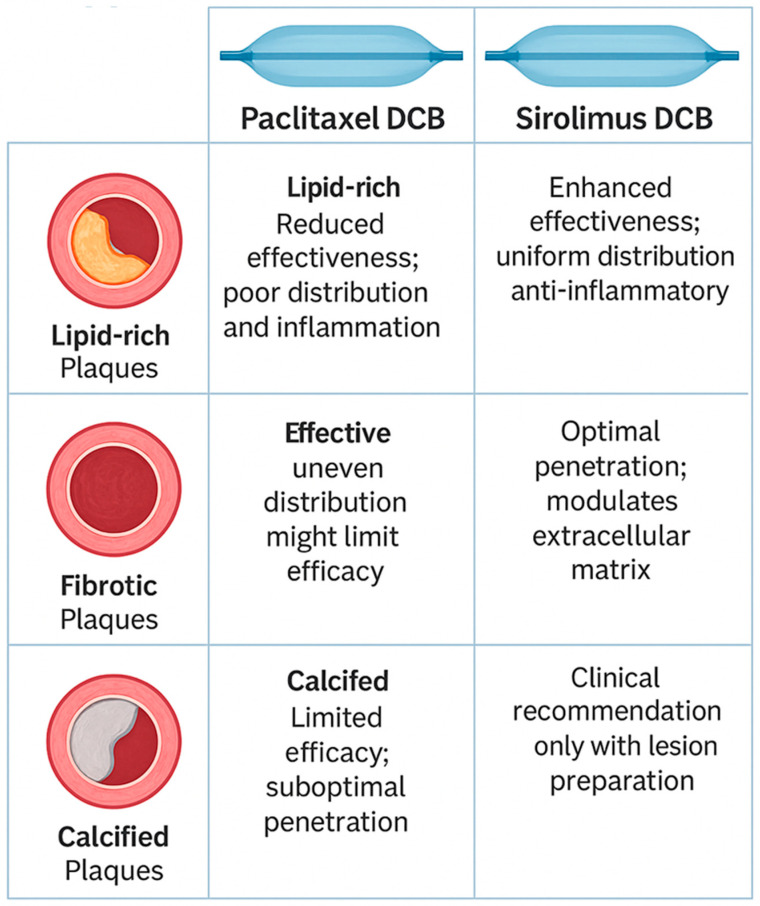
Infographic illustrating the effects of sirolimus-coated balloons across lipid-rich, fibrotic, and calcified plaques.

Clinical experience and early comparative data suggest distinct roles for PEBs and SEBs in clinical practice. While PEBs have demonstrated robust efficacy in restenotic lesions and stable fibrocalcific plaques, SEBs may be advantageous in inflammatory, lipid-rich, or otherwise vulnerable plaques, potentially reducing late restenosis and improving vessel healing. Nonetheless, definitive randomized trials comparing clinical outcomes across distinct plaque morphologies remain limited, highlighting a critical need for further investigation to guide individualized decision making in PCI [39,40,41,42] (Table 3, Figure 4).

**Figure 5:**
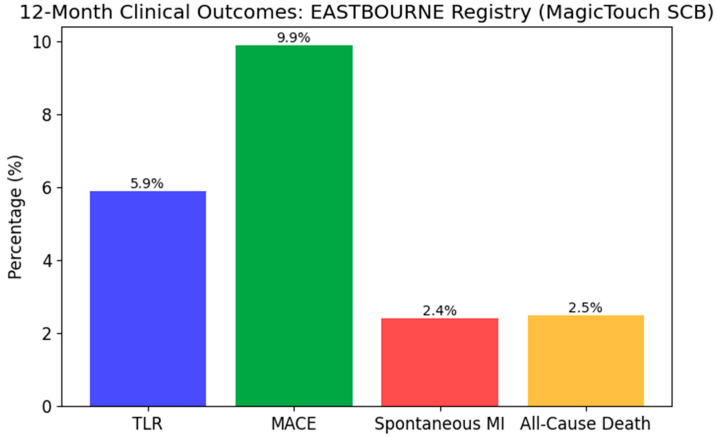
Graphical representation of clinical outcomes from the EASTBOURNE Registry (MACE, spontaneous MI, all-cause death, and TLR).

In conclusion, careful consideration of plaque morphology should guide the choice between paclitaxel- and sirolimus-eluting balloons (Figure 5). Paclitaxel may retain its role in straightforward, stable plaque scenarios, whereas sirolimus holds promise for addressing the complexity of unstable or inflammatory plaque lesions, potentially offering broader therapeutic benefits in such contexts.

Table 4 summarizes pivotal contemporary studies evaluating the efficacy of drug-coated balloons (DCBs), either paclitaxel- or sirolimus-coated, in comparison to drug-eluting stents (DESs) or other PCI strategies. For instance, the BASKET-SMALL 2 trial demonstrated noninferiority of paclitaxel-DCB to second-generation DES in small vessel coronary artery disease, with comparable rates of major adverse cardiovascular events (MACE). Similarly, PEPCAD China ISR and RIBS IV supported the use of DCBs in in-stent restenosis, showing reduced late lumen loss (LLL) and favorable target lesion revascularization (TLR) outcomes. Studies like EASTBOURNE further confirmed the safety and feasibility of sirolimus-coated balloons across broader populations. While sirolimus DCBs are newer and supported by less extensive trial data, emerging evidence from BIOLUX and PICCOLETO II suggests they may offer similar efficacy with potential advantages in delayed drug release and endothelial healing. These findings suggest DCBs, particularly in select lesion types and patient profiles, are becoming viable alternatives to DES in both de novo and restenotic lesions. Their value is especially pronounced where minimizing stent use and shortening dual antiplatelet therapy are desirable.

While theoretical and mechanistic differences between plaque types and drug properties are discussed, limited clinical data currently stratify DCB outcomes by plaque morphology (e.g., fibrotic vs. lipid-rich vs. calcified). Future trials integrating intravascular imaging (OCT/IVUS) with outcome tracking by lesion type are warranted.

While this review outlines theoretical and pharmacologic considerations for DCB selection based on plaque type, direct comparative clinical data stratified by morphology remain sparse. As such, these proposals serve as a hypothesis-generating framework until validated by future stratified studies.

## 10. Clinical Scenarios Where DCB Use May Be Avoided

**Figure 6:**
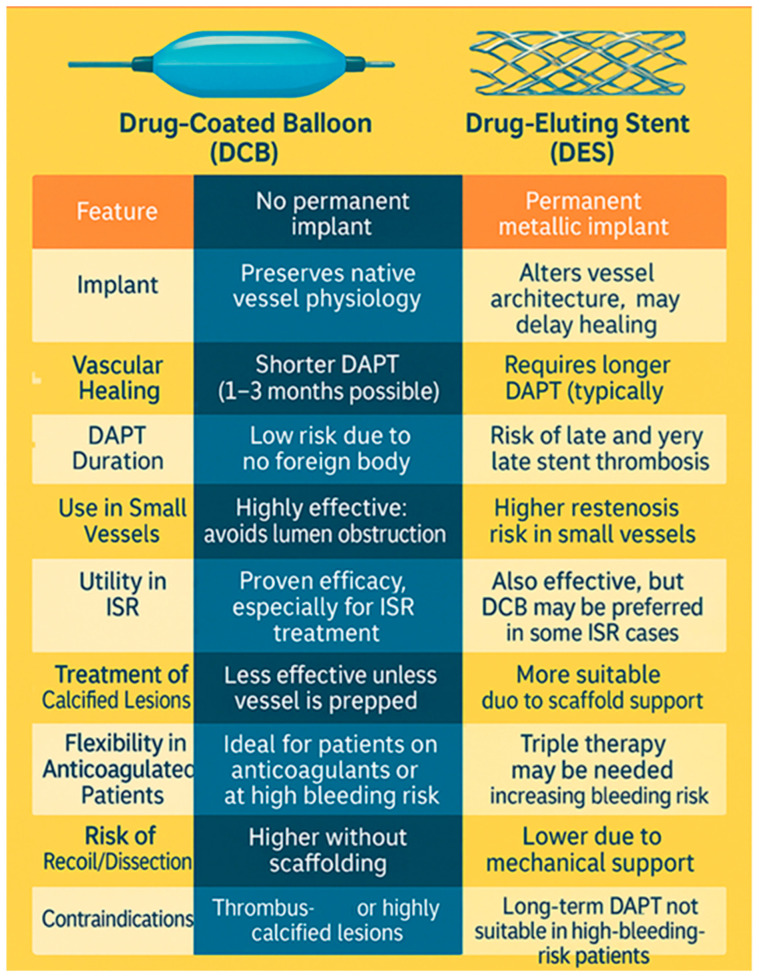
Graphical picture with DCB and DES discussing the key advantages and disadvantages for both these PCI strategies.

While drug-coated balloons (DCBs) offer important advantages, there are specific clinical situations where their use may be less appropriate or even contraindicated. Heavily calcified lesions often require plaque modification techniques, such as atherectomy, to ensure effective drug penetration; without adequate preparation, DCB efficacy may be limited (Figure 6). Thrombus-rich lesions also pose a challenge, as the presence of thrombus increases the risk of distal embolization and can impair drug transfer to the vessel wall. In cases where mechanical support is needed, such as long, unstable lesions prone to recoil or dissection, the absence of a permanent scaffold with DCB use may result in poor clinical outcomes, and a stent may be more appropriate. Additionally, in complex bifurcation lesions, where maintaining side branch patency is critical, DCBs alone may not provide sufficient control or protection, making stenting a more reliable strategy.

## 11. Clinical Implications and Decision-Making Framework

The choice of drug-coated balloon in percutaneous coronary intervention should be guided not only by the lesion setting, such as de novo disease or in-stent restenosis, but also by the underlying plaque morphology, which can significantly affect drug delivery, absorption, and vascular response. Based on available mechanistic and clinical data, sirolimus-coated balloons may be more suitable for lipid-rich and inflammatory plaques due to their uniform tissue distribution, cytostatic action, and strong anti-inflammatory properties. These characteristics help stabilize vulnerable plaques, reduce macrophage-driven inflammation, and promote vessel healing.

In contrast, paclitaxel-coated balloons appear to be particularly effective in fibrotic and stable plaques, where their potent antiproliferative effects can prevent neointimal hyperplasia and restenosis. This is especially relevant in the context of in-stent restenosis and small vessel disease, where PCBs have demonstrated consistent clinical benefit.

Calcified plaques remain challenging for both drug delivery and vascular remodeling. In these lesions, pre-treatment strategies such as scoring balloons or atherectomy may be necessary to improve drug penetration. While both PCB and SCB options have limitations in heavily calcified lesions, SCBs may offer theoretical advantages in calcified plaques with active inflammation due to their better tissue diffusion and anti-inflammatory action.

Taken together, tailoring DCB therapy based on plaque morphology offers a practical and potentially impactful framework for clinical decision making. Prospective studies are warranted to validate this approach and better define the optimal use of DCB technologies across diverse lesion types.

## 12. Conclusions

The evolution of drug-coated balloon technology has provided an effective stent-free alternative for treating coronary artery disease, offering unique advantages over traditional drug-eluting stents. While paclitaxel- and sirolimus-coated balloons both effectively prevent restenosis, their pharmacokinetics, plaque interactions, and clinical efficacy differ, influencing their optimal use in PCI.

Paclitaxel demonstrates rapid tissue absorption and facilitates positive vascular remodeling, making it particularly effective in fibrotic plaques. Its poor penetration in calcified plaques presents a limitation, requiring vessel preparation techniques to enhance drug delivery. Conversely, sirolimus exhibits more homogeneous tissue distribution and sustained drug release, leading to superior plaque stabilization and reduced inflammation, particularly in unstable, lipid-rich, and inflammatory plaques.

Recent randomized trials and meta-analyses have demonstrated non-inferiority of SCBs compared to PCBs in in-stent restenosis, with some studies suggesting potential advantages in long-term vascular healing. Additionally, emerging evidence supports the role of DCBs in de novo lesions, particularly in small vessel disease, where they show comparable outcomes to DES while avoiding the complications of permanent metallic scaffolds.

Moving forward, further long-term studies and head-to-head comparisons are necessary to refine patient and lesion selection criteria for DCB therapy. Additionally, advancements in plaque modification techniques and DCB formulations may further optimize outcomes, particularly in challenging lesion subsets such as heavily calcified arteries. Ultimately, a personalized approach to PCI, guided by plaque morphology, intravascular imaging, and patient-specific factors, will help maximize the benefits of DCB therapy in contemporary interventional cardiology.

Although current evidence lacks robust stratification by plaque type, this review provides an interpretive approach that integrates drug-specific effects with lesion morphology. This novel perspective may inform individualized PCI strategies while underscoring areas where prospective, imaging-guided trials are needed.

## Figures and Tables

**Table 1:** Comparison of drug carriers and delivery characteristics in paclitaxel- and sirolimus-coated balloons.

Carrier	Drug Type	Industry Example	Solubility	Inflation Time for Drug Transfer	Mechanism	Clinical Impact
**Iopromide**	Paclitaxel	SeQuent Please DCB [22]	Hydrophilic	30–60 s	Enhances rapid drug transfer but may cause distal embolization	Effective in restenosis reduction but has potential embolization risk
**BTHC** **(Butyryl-trihexyl-citrate)**	Paclitaxel	Pantera Lux DCB [23]	Lipophilic	45–60 s	Increases retention in tissues, reducing drug washout	Provides prolonged drug effect but may delay healing
**Urea**	Paclitaxel	Prevail DCB [24]	Hydrophilic	30 s	Improves drug diffusion but allows faster washout	Rapid transfer but shorter tissue retention
**Shellac**	Sirolimus	Magic Touch DCB [25]	Hydrophobic	60 s	Forms a controlled-release layer for sustained retention	Reduces inflammation and enhances endothelial recovery
**Urea**	Sirolimus	Selution SLR DCB [26]	Hydrophilic	30 s	Allows rapid drug diffusion but may lead to shorter retention	Effective but may have shorter therapeutic duration
**BTHC** **(Butyryl-trihexyl-citrate)**	Sirolimus	Virtue DCB [27]	Lipophilic	45–60 s	Enhances adhesion and retention, ensuring extended release	Prolonged therapeutic action, useful for complex lesions
**Phospholipid-based**	Sirolimus	Experimental SCBs 27]	Amphiphilic	Variable	Mimics cell membranes to improve sirolimus absorption	Sustained drug release with improved vessel healing

**Table 2:** Summary of key DCB clinical studies.

Study Name	Study Design	Number of Patients	Lesion Type	Intervention Arms	Main Findings	Follow-Up
PEPCAD II	RCT	131	ISR	DCB vs. DES	DCB non-inferior to paclitaxel-DES in ISR	12 months
BIOLUX RCT	RCT	302	ISR	DCB vs. DES	DCB showed comparable efficacy to DES	12 months
EASTBOURNE	Prospective Registry	2111	All-comers	SCB	SCB showed safety and efficacy in large-scale use	12 months
BASKET-SMALL 2	RCT	758	Small vessels	DCB vs. DES	DCB non-inferior to DES in small vessels	12 months
FIRE	RCT	1500	Complex PCI in elderly	DCB vs. DES	DCB associated with lower bleeding, non-inferior ischemic outcomes	12 months
RCT	RCT	232	Small vessel CAD	PCB vs. DES	PCB DCB non-inferior to everolimus DES for late lumen loss	6 months

**Table 3:** Comparison of paclitaxel and sirolimus drug-eluting balloons across coronary plaque types.

Aspect	Paclitaxel	Sirolimus
**Mechanism of Action**	Non-uniform (crystalline, lipophilic), potential for distal embolization	Cytostatic and immunosuppressive; inhibits mTOR pathway and cell-cycle transition (G1/S), reducing proliferation and inflammation
**Distribution in Vessel Wall**	Non-uniform distribution, potential embolization	Homogeneous distribution, lower embolization risk
**Stable Fibrocalcific Plaque**	Preferred; uneven distribution might limit efficacy	Effective; uniform penetration, inflammatory control
**Lipid-rich Plaque**	Reduced effectiveness due to poor distribution and inflammation	Enhanced effectiveness; uniform distribution, anti-inflammatory properties
**Vulnerable Plaque (thin-cap fibroatheroma)**	Limited efficacy; increased inflammation risk	Superior; stabilizes plaque due to anti-inflammatory effects
**Plaque with Thrombus**	Limited; risk of inflammation and embolization	Preferred; anti-inflammatory and stabilizing properties
**Clinical Recommendations**	Stable lesions, restenosis, fibrocalcific plaques	Unstable, inflammatory, lipid-rich, thrombotic lesions

**Table 4:** Summary of studies on sirolimus- and paclitaxel-coated balloons.

	Combined Analysis (2022)	Randomized Trial (2024)	EASTBOURNE Registry (2023)	BIOLUX Trial (2024)
**Population**	101 patients with DES-ISR	70 patients with de novo lesions	2123 patients (2440 lesions) with CAD	229 patients with ISR
**Inclusion Criteria**	DES in-stent restenosis	De novo coronary lesions suitable for PCI	All-comer CAD, including small vessels and ISR	ISR in BMS or DES
**Exclusion Criteria**	Not explicitly stated	Not explicitly stated	Not explicitly stated	Not explicitly stated
**Intravascular Imaging**	Not specified	OCT-guided balloon sizing	Not specified	Not specified
**Primary Objectives**	In-segment late lumen loss (6 months)	Net lumen diameter gain at 6 months	Target lesion revascularization (12 months)	In-stent late lumen loss (6 months)
**Secondary Objectives**	MACE, TLR, stent thrombosis (12 months)	MACE, TLR (12 months)	MACE, spontaneous MI, cardiac/all-cause death	Target lesion failure (18 months)
**Key Conclusions**	SCB and PCB show similar angiographic and clinical outcomes	SCB non-inferior to PCB in de novo lesions	SCB safe and effective with low TLR and MACE rates	Paclitaxel DCB comparable to sirolimus DES in ISR lesions

## Abbreviations

The following abbreviations are used in this manuscript:

BMS	Bare Metal Stent
CAD	Coronary Artery Disease
DAPT	Dual Antiplatelet Therapy
DCB	Drug-Coated Balloon
DES	Drug-Eluting Stent
ISR	In-Stent Restenosis
IVUS	Intravascular Ultrasound
LCBI	Lipid Core Burden Index
LLL	Late Lumen Loss
MACE	Major Adverse Cardiac Events
OCT	Optical Coherence Tomography
PCB	Paclitaxel-Coated Balloon
PCI	Percutaneous Coronary Intervention
SCB	Sirolimus-Coated Balloon
TLF	Target Lesion Failure
TLR	Target Lesion Revascularization
